# Suppression of Pepper Root Rot and Wilt Diseases Caused by *Rhizoctonia solani* and *Fusarium oxysporum*

**DOI:** 10.3390/life12040587

**Published:** 2022-04-14

**Authors:** Mohamed Kamal El-kazzaz, Kamal Elsayed Ghoneim, Mohamed Khaled Mohamed Agha, Asmaa Helmy, Said I. Behiry, Ahmed Abdelkhalek, Muhammad Hamzah Saleem, Abdulaziz A. Al-Askar, Amr A. Arishi, Mohsen Mohamed Elsharkawy

**Affiliations:** 1 Department of Agricultural Botany, Faculty of Agriculture, Kafrelsheikh University, Kafr El-Sheikh 33516, Egypt; mohamed.elkazaz@agr.kfs.edu.eg (M.K.E.-k.); kamal.ghonaim@agr.kfs.edu.eg (K.E.G.); 2 Plant Protection Department, Desert Research Center, El-Matareya, Cairo 11753, Egypt; virusb007@yahoo.com (M.K.M.A.); asmaa_daif22@yahoo.com (A.H.); 3 Agricultural Botany Department, Faculty of Agriculture (Saba Basha), Alexandria University, Alexandria 21531, Egypt; said.behiry@alexu.edu.eg; 4 Plant Protection and Biomolecular Diagnosis Department, ALCRI, City of Scientific Research and Technological Applications, New Borg ElArab City, Alexandria 21934, Egypt; 5 MOA Key Laboratory of Crop Ecophysiology and Farming System Core in the Middle Reaches of the Yangtze River, College of Plant Science and Technology, Huazhong Agricultural University, Wuhan 430070, China; saleemhamza312@webmail.hzau.edu.cn; 6 Department of Botany and Microbiology, College of Science, King Saud University, P.O. Box 2455, Riyadh 11451, Saudi Arabia; aalaskara@ksu.edu.sa; 7 School of Molecular Sciences, The University of Western Australia, Perth, WA 6009, Australia; 22650755@student.uwa.edu.au

**Keywords:** *Fusarium oxysporum*, *Rhizoctonia solani*, plant growth promoting fungi, pepper, *Trichoderma longibrachiatum*, *Paenibacillus polymyxa*

## Abstract

Pepper is vulnerable to soil-borne fungal pathogens such as *Rhizoctonia solani* and *Fusarium oxysporum*. The potential of beneficial rhizosphere microorganisms to control *R. solani* and *F. oxysporum* f.sp. *capsici* was evaluated in pepper plants. *Paenibacillus polymyxa* and *Trichoderma longibrachiatum* were isolated from rhizospheric soil samples of healthy pepper plants. In vitro, both isolates caused clear reductions in the radial growth of root rot and wilt pathogens. Scanning electron microscopy displayed lysis and abnormal shape of the pathogens in dual cultures with *P. polymyxa* and *T. longibrachiatum*. The incidence and severity of root rot and wilt diseases were significantly reduced in pepper plants treated with the growth-promoting fungi (PGPF isolates; *Fusarium equiseti* GF19-1, *Fusarium equiseti* GF18-3, and *Phoma* sp. GS8-3), *P. polymyxa*, or *T. longibrachiatum* in comparison to the control. Moreover, the induction treatments led to increased pepper growth compared with their control. The defense related gene (*CaPR4*) expression was shown to be significantly higher in the treated plants than in the control plants. In conclusion, the antagonistic isolates (*P. polymyxa* and *T. longibrachiatum*) and PGPF isolates have a clear impact on the prevention of root rot and wilt diseases in pepper plants incited by *R. solani* and *F. oxysporum* f.sp. *capsici.* The expression of the *CaPR4* gene added to the evidence that PGPF isolates generate systemic resistance to pathogen infections.

## 1. Introduction

Pepper (*Capsicum annuum* L.) is a commonly cultivated crop, from the family Solanaceae, in Egypt and all over the world. In agricultural production, soil-borne pathogens are the main problems causing reductions in crop yield and quality. The most dreaded diseases of vegetables are root rot, damping-off, charcoal rot, and wilt caused by *Rhizoctonia solani*, *Alternaria solani*, *F. oxysporum* f.sp. *capsici*, *Sclerotium rolfsii*, *Macrophomina phaseolina*, and *Pythium* spp. [1]. Pepper is susceptible to several soil-borne pathogenic fungi, causing severe plant death and large losses all over the world [2]. However, *Fusarium* wilt is known to be one of the most important devastating and harmful diseases affecting pepper plants. Pre- and post-emergence damping-off, root rot, wire stem, seed decay, and hypocotyl or tap root with necrotic spots, all of these symptoms are caused by *R. solani* at multiple growth stages [3]. *F. oxysporum* and *R. solani* were the most common isolates from root-rooted and wilted pepper plants. Pathogenicity tests on the isolated pathogens from the infected pepper plants demonstrated that plants were infected with *F. oxysporum* f.sp. *capsici* and *R. solani* [4]. Likewise, plants infected with *F. oxysporum f.sp. capsici* showed damping-off and vascular wilt symptoms [5].

Biological control could be a successful strategy for managing diseases and feasible pepper production. Biocontrol agents (BCAs) control plant pathogens through several mechanisms, such as fungistasis, antibiosis, hyperparasitism, induced systemic resistance, and modification of the rhizosphere [6]. Plant growth-promoting fungi (PGPF) are a group of non-pathogenic soil-inhabiting fungi that improve several growth characters after treatments in numerous plants [6,7]. Typically, PGPF are useful for the management of soil-borne fungi [5]. Beneficial bacterial and fungal isolates (*Pseudomonas fluorescens*, *Bacillus subtilis*, *Fusarium equiseti*, *Penicillium simplicissmum*, *Trichoderma viride*, and *T. harzianum*) were effective in soil-borne disease control and enhancement of plant growth [2,5,6]. The non-pathogenic isolates of *F. moniliforme*, *F. oxysporum*, *F. solani*, and *F. merismoides* reduced wilt incidence in tomato [8].

Pathogen infection is associated with induced genes encoding pathogenesis-related (PR) proteins. Plants have evolved a variety of defensive mechanisms to restrict pathogen attack, including biochemical, physiological, molecular, and cellular processes and barriers, as well as inducible defense response [5,6,7,9]. In PGPF treated onion plants, accumulation of peroxidase and polyphenol oxidase were stimulated in comparison to the control infected with *Sclerotium cepivorum*. Additionally, exalted expressions of defense-related genes, *PR1* and *PR2*, have been described in plants treated with PGPF [9]. Various defense mechanisms against pathogen infection were explored in chili plants [10]. The *CaChi2* gene was involved in resistance to *F. oxysporum* f.sp. *capsici* in chili plants. The objective of this research was to test the efficiency of certain bioagents in controlling pepper root rot and wilt diseases under greenhouse conditions. Additionally, the activities of defense enzymes and expression of defense genes were evaluated.

## 2. Materials and Methods

### 2.1. Isolation of the Causal Pathogens and the Rhizosphere Microorganisms

Root samples of diseased pepper plants were isolated and identified in our previous study [4]. Samples of pepper plants infected with the pathogens were gathered from several Egyptian governorates, cleaned with tap water, and sterilized by soaking them in 5% sodium hypochlorite for 2 min before being rinsed with sterile water. Potato dextrose agar medium (PDA) was used for the incubation of thin slices in Petri plates for five days at 26 °C. The hyphal tips of each of the newly emerging fungus were transferred to PDA plates where they were purified based on morphological and molecular characteristics. The isolates *Fusarium equiseti* GF19-1, *F. equiseti* GF18-3, and *Phoma* sp. GS8-3 were used against *Bean yellow mosaic virus* [11]. The suppression effects of these isolates were tested and compared with the efficiency of new and novel bioagents against *R. solani* and *F. oxysporum* f.sp. *capsici.*

Several bioagents, isolated from the rhizosphere of healthy pepper roots, were gathered from different locations, i.e., Kafr El-Sheikh, El-Behira, North Sinai, and Alexandria governorates. The serial dilution method was utilized for isolation. Identification of bacterial isolates was carried out depending on cultural, morphological, and biochemical properties [12], while fungal isolates were identified following the methods stated by Melo and Faull [13]. Moreover, 16S rRNA and internal transcribed spacer (ITS) sequencing were utilized for molecular identification. DNA was extracted by using the protocol of GeneJet genomic DNA purification Kit (Thermo K0721) as described by Elsharkawy and El-Sawy (2019). PCR was performed using the primer F: AGA GTT TGA TCC TGG CTC AG, R: GGT TAC CTT GTT ACG ACT T, Maxima Hot Start PCR Master Mix (Thermo Fisher Scientific, Waltham, MA USA) (Thermo K1051) and GeneJET™ PCR Purification Kit (Thermo Fisher Scientific, Waltham, MA USA) (Thermo K0701) following manufacturer protocol. Sequencing was done using ABI 3730xl DNA sequencer as described by Elsharkawy and El-Sawy [12].

### 2.2. Effect of Bacterial and Fungal Antagonists on Root Rot and Wilt Pathogens In Vitro

For antagonistic bacterial isolates, PDA plates were inoculated in the center with agar discs (5 mm) bearing mycelium of 7-days old cultures of *R. solani* (R) and *F. oxysporum* f.sp. *capsici* (F). Plates were concurrently inoculated by bacteria (four isolates per dish). Non-inoculated plates served as control. Plates were kept at 19–22 °C until the control treatment was fully covered with PDA. The diameter of inhibition zone was measured, and the relative power of antibiosis (RPA) was determined using the formula: RPA = Z/C [14]. Z: The diameter of the inhibition zone. C: The diameter of spotted antagonistic isolate.

For fungal antagonists, the dual culture technique was used for testing the antagonistic activity of fungal isolates. PDA plates were inoculated with discs (5 mm) of the pathogenic fungi (one-week old cultures), and on the opposite side, the discs of the antagonistic fungi were placed. Plates containing the pathogens alone have been considered as control. Plates were kept at 18–20 °C until the full growth in control plates. The degree of antagonisms was determined as elucidated by Bell et al. [15,16].

### 2.3. Microscopic Examination

Scanning electron microscopy (SEM) has been utilized to evaluate the effects of *Paenibacillus polymyxa* and *Trichoderma longibrachiatum* (the most effective bio-agents in the experiments compared with other bioagents in vitro) on the growth of *F. oxysporum* f.sp. *capsici* and *R. solani* [12]. The samples were immersed for fixation in a modified Karnovsky [17] solution (2.5% buffered glutaraldehyde +2% paraformaldehyde in 0.1 M sodium phosphate buffer pH 7.4). The tissues have been incubated overnight at 4 °C followed by washing three times in 0.1 M sodium phosphate buffer and 0.1 M sucrose. The tissues were post-fixed in 2% sodium phosphate with osmium tetroxide pH 7.4 for 90 min. Sodium phosphate buffer pH 7.4 (0.1 M) was used for washing the samples for three times. Serial dilutions of ethanol were utilized for the dehydration of samples. The samples were put in a critical point drying and specimens were coated with gold-palladium membranes and observed in a Jeol JSM-6510L.V SEM. The microscope was operated at 30 KV in EM Unit, Mansoura University, Egypt.

### 2.4. Effect of Fungal and Bacterial Isolates on Wilt and Root Rot Diseases under Greenhouse Conditions

#### 2.4.1. Preparation of Bacterial Inoculum

Inoculum of the antagonistic isolate was prepared by growing on nutrient broth media in conical flasks (500 mL) at 28 °C for 5 days using shaking incubator (100 rpm). Cell suspension was diluted and adjusted to 10^8^ cfu/mL of *P. polymyxa.*

#### 2.4.2. Preparation of Fungal Inoculum

Inocula of the isolates *T. longibrachiatum*, *F. equiseti* GF19-1, *F. equiseti* GF18-3, and *Phoma* sp. GS8-3 were prepared by growing them on potato dextrose broth medium (PDB) and incubated at 28–30 °C for 10 days. Spore suspensions of the isolates were counted and adjusted to 2 × 10^6^ spore mL^−1^ [18].

#### 2.4.3. Plant Growth Conditions

Pot experiments were carried out during two consecutive seasons for studying the effect of the five isolates on root-rot and wilt diseases of pepper. Plastic pots (24 cm in diameter) were filled with autoclave sterilized sandy loam soil infested with one of the two pathogens, *F. oxysporum* f.sp. *capsici* or *R. solani*, at the rate of 2% (*w*/*w*, inoculum/soil). Inoculum of each pathogen was mixed separately with soil. Infested pots were irrigated and kept for 5 days before transplanting [4]. Pots were planted with pepper seedlings (3 seedlings per pot).

#### 2.4.4. Induction Treatments

a-Seed treatment

Pepper seeds were treated by soaking in 10 mL of the bacterial suspension (10^8^ cfu/mL) or fungal spore suspension (2 × 10^6^ spore/mL) for 2 h before planting in seedling trays [19]. Seeds were air-dried in sterile petri plates. Three seeds were sown in each well of the tray. Untreated pepper seeds were utilized as a control.

b-Seedling treatment

A seedling tray was filled with potting soil and sown with sterilized pepper seeds (using 5% sodium hypochlorite). The 19-d-old seedlings, raised in the tray, were soaked in the suspension of each isolate for 2 h and transferred to small pots (5 cm in diameter) filled with the potting soil. Twenty days later, the seedlings were transplanted in pots artificially infested with the pathogen (2% *w*/*w*). As a control treatment, autoclaved PDB was used instead of bioagent suspensions.

#### 2.4.5. Disease Assessment

Survived seedlings were removed, washed, and scored for *R. solani* as described by O’Sullivan and Kavanagh [20]. For the assessment of Fusarium wilt disease severity, foliar symptoms were evaluated as explained by Horinouchi et al. [5]. Discoloration severity of vascular tissues was assessed as described by Horinouchi et al. [5]. Plant growth parameters such as plant height, number of leaves, and shoot (fresh and dry) weight were determined [6].

#### 2.4.6. Assessment of Defense Enzymes and Total Phenol Contents

Using a liquid nitrogen-cooled mortar and pestle, 1 g of freshly inoculated pepper leaves (3 days after inoculation) were crushed into powder. Subsequently, the obtained powder was macerated for 30 s and homogenized with sodium phosphate buffer (3 mL, pH 6.8, 0.01 M). The filtrates were centrifuged (15 min, 6000 rpm, 4 °C) after filtering the triturated tissues through four layers of cheese cloth. A sample of the clear supernatant was collected for enzyme extraction.

Peroxidase and polyphenol oxidase activities in the collected samples were estimated (min^−1^ g^−1^) by spectrophotometric analysis [9]. Estimation of total phenol contents (expressed as milligram per gram of sample) was determined [21].

#### 2.4.7. Molecular Investigation of Pathogenesis-Related Genes Expression

Leaves were harvested at 2 days after pathogen inoculation. RNA Purification Kit (Thermo Fisher Scientific, Waltham, MA USA) was used to extract RNA. The extracted RNA was converted to complementary DNA (cDNA) using revert Aid H minus reverse transcriptase. Real-time PCR with SYBR Green was used to measure the expression of mRNAs of the target gene (*CaPR4*, a gene associated with defense response and cell death), with *CaActin* as an internal reference as described by Elsharkawy and El-Khateeb [9].

#### 2.4.8. Re-Isolation Frequency

At 7 weeks after planting, root colonization of pepper plants with PGPF isolates was assessed for both PGPF and control treatments. The roots were picked from random plants and cleaned three times using sterile-distilled water before drying with a paper towel. Afterwards, they were sliced into 1-cm pieces and placed onto PDA. The frequency of PGPF was assessed as described by Elsharkawy et al. [6].

### 2.5. Statistical Analysis

The experimental data from trials were combined for analysis of variance. The experiments had been done three times. Means were separated by Fisher’s LSD test using XLSTAT Software (Addinosoft).

## 3. Results

### 3.1. Isolation and Screening of Fungal and Bacterial Antagonists In Vitro

**Figure 1:**
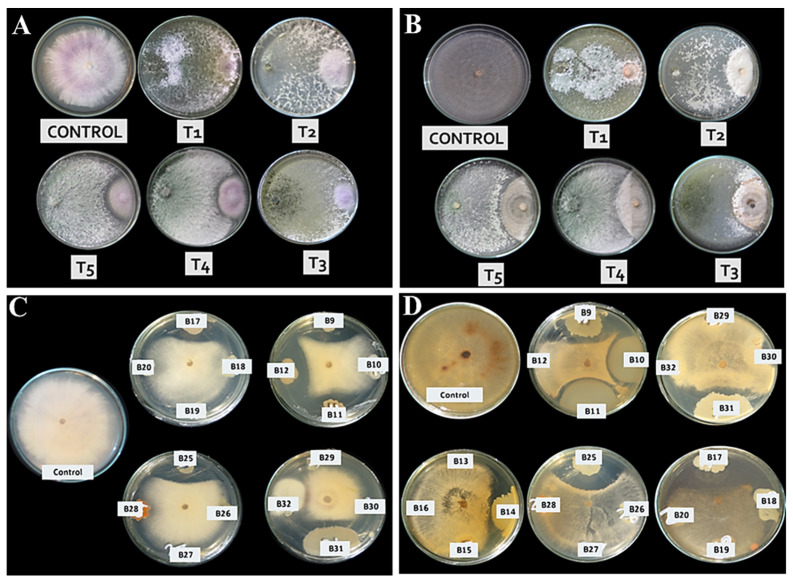
Degree of antagonism of Trichoderma and bacteria isolates against Fusarium oxysporum f.sp. capsici (**A**,**C**) and Rhizoctonia solani (**B**,**D**). *Trichoderma longibrachiatum* is T1, *T. aureoviride* is T2, *T. hamatum* is T3, *T. harzianum* 1 is T4, *T. harzianum* 2 is T5, *B. subtilis* is B17, *B. subtilis* is B9, *B. thuringiensis* is B11, *B. subtilis* is B12, *B. subtilis* is B31, and Paenibacillus polymyxa is B25.

Five fungal isolates of *Trichoderma* spp. (T1, T2, T3, T4, and T5) as well as 32 bacterial isolates were isolated from different rhizosphere samples of healthy pepper plants. The isolate *T. longibrachiatum* (T1) proved to have the highest antagonistic effect on the pathogens (Figure 1A,B and Table 1). Among 32 bacterial isolates, six bacterial isolates had significant antagonistic effects on the linear growth of *F. oxysporum* f.sp. *capsici* in Petri dish (Table 2). The highest inhibitory spectrum between the isolates was *P. polymyxa* (B25) (Figure 1C). Six bacterial isolates had significant antagonistic effects on the linear growth of *R. solani.* The highest inhibitory spectrum of the isolates was *P. polymyxa* (B25) (Figure 1D).

### 3.2. Identification of the Most Efficient Fungal and Bacterial Isolates

**Figure 2:**
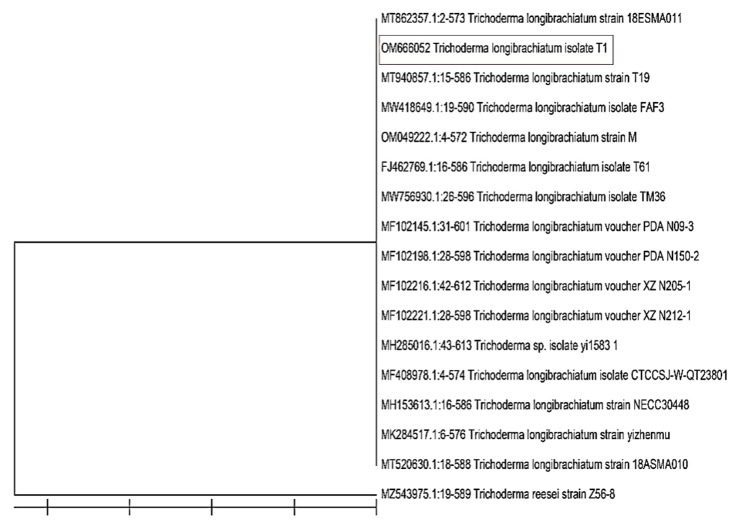
Neighbor-joining phylogenetic dendrogram showing the position of *Trichoderma longibrachiatum* among phylogenetic neighbors.

**Figure 3:**
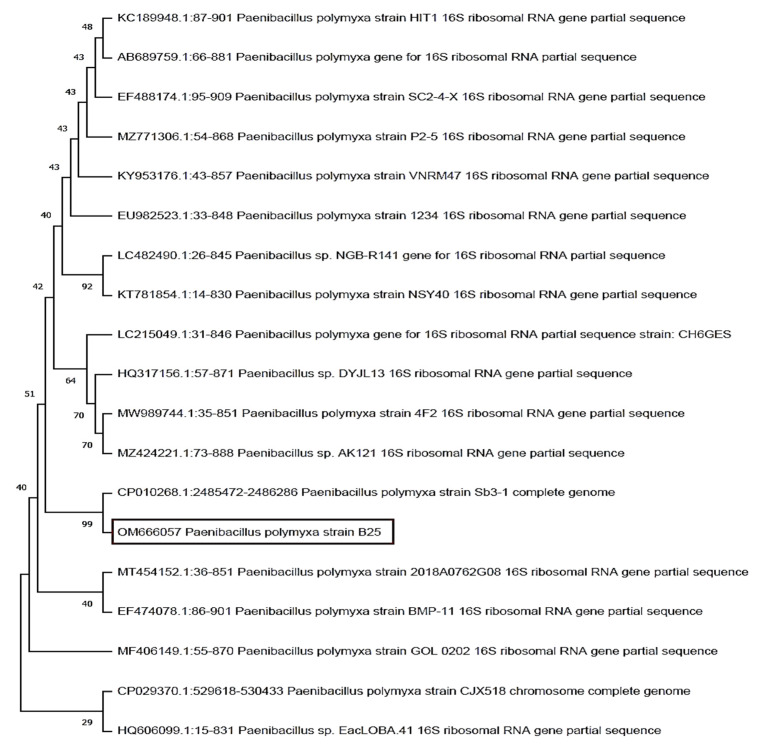
Phylogenetic dendrogram showing the position of *Paenibacillus polymyxa* strain B25 among phylogenetic neighbors.

The phylogenetic tree exhibited that the *Trichoderma* isolate T1 was strongly related to the species *longibrachiatum.* It showed the highest sequence similarities with *T. longibrachiatum* (GenBank accession number OM666052) (Figure 2). On the other hand, the phylogenetic tree showed the relation between *Paenibacillus* isolate and the related bacterial species (Figure 3). It can be clearly seen that the *Paenibacillus* isolate B25 was highly related to the species *polymyxa*. It showed the highest sequence similarities with *P. polymyxa* strain B25 (GenBank accession number OM666057).

### 3.3. Scanning Electron Microscopy (SEM)

**Figure 4:**
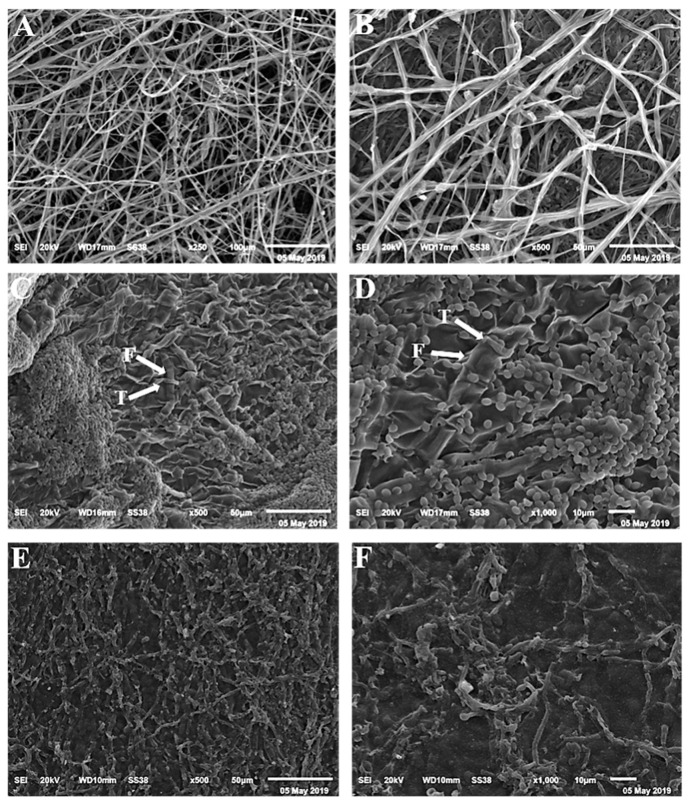
Scanning electron microscopy (SEM) for *Fusarium oxysporum* f.sp. *capsici.* The growth of wilt pathogen in control (**A**,**B**), dual culture with *Trichoderma longibrachiatum* (**C**,**D**), and dual culture with *Paenibacillus polymyxa* (**E**,**F**).

The SEM examination showed a complete fungal growth of *F. oxysporum* f.sp. *capsici* (Figure 4A,B). Full coalescence was developed from the mycelia in their ideal form in control. However, the dual culture with *T. longibrachiatum* showed morphological anomaly and coiling of *F. oxysporum* f.sp. *capsici* (Figure 4C,D). At the same time, *P. polymyxa* demonstrated morphological abnormality such as degradation and lysis in the fungal mycelia (Figure 4E,F).

**Figure 5:**
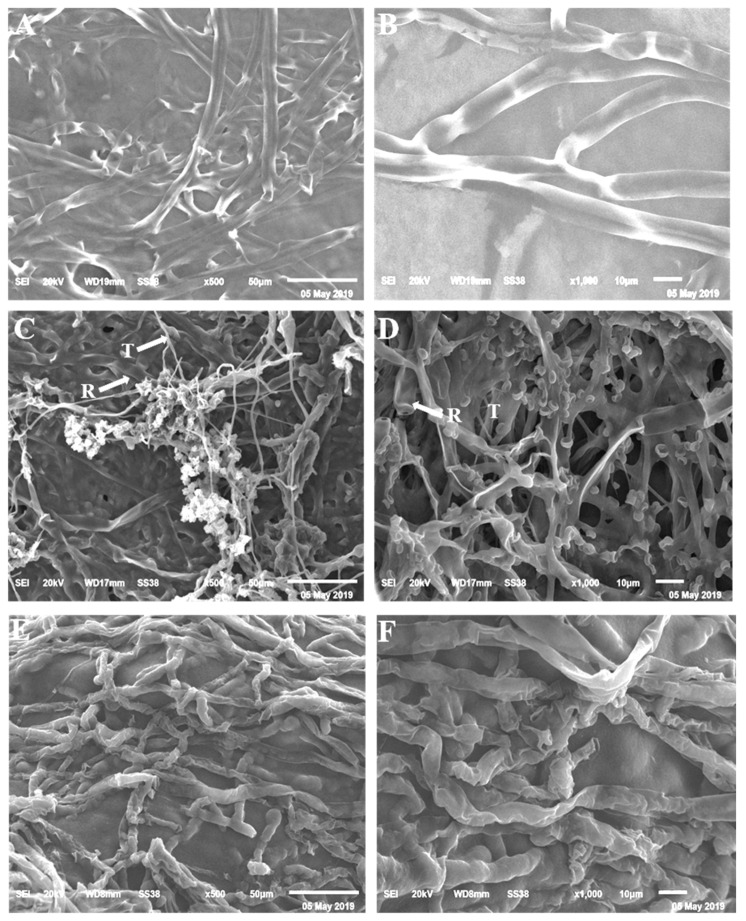
Scanning electron microscopy (SEM) for *R. solani.* The growth characters of root rot pathogen in control (**A**,**B**), dual culture with *Trichoderma longibrachiatum* (**C**,**D**), and dual culture with *Paenibacillus polymyxa* (**E**,**F**).

Typical morphological characteristics of *R. solani* was observed using SEM (Figure 5A,B). In contrast, overgrowth and lysis were observed in dual cultures of *R. solani* and *T. longibrachiatum* (Figure 5C,D), while lysis and atrophy were observed in dual cultures of *R. solani* and *P. polymyxa* (Figure 5E,F).

### 3.4. Effect of Antagonistic and PGPF Isolates on F. oxysporum f.sp. capsici and R. solani under Greenhouse Conditions

Table 3 showed that all the examined bioagents significantly reduced disease incidence and severity of wilt and root rot in comparison to their control as a result of seed and seedlings treatments. The most successful treatment for *Fusarium* wilt severity was GF19-1 as a seedling treatment, which gave disease severity 27.8% and 25.0% in the first and second seasons, respectively. The most helpful treatments for disease incidence caused by *F. oxysporum* f.sp. *capsici* were GF19-1 and GF18-3 as seedling treatments, which gave the same disease incidence in both seasons (27.8%). However, the most successful treatment on *R. solani* was GF18-3 as seedling treatment, which gave a disease severity percentage of 27.77% in both seasons. Likewise, the most useful treatment for disease incidence of *Rhizoctonia* root rot was GF18-3 as seedling treatment, which gave a disease incidence of 27.8%. Discoloration severity was estimated at the end of the experiment. Discoloration severity was reduced in the treatments with GF19-1 and GF18-3 as seedling treatments recording 14.8% in the second season (Table 4).

#### 3.4.1. Effect of Different Bioagents on Some Plant Growth Parameters

A significant increase in plant height, number of leaves, fresh and dry weights (g)/plant was verified due to biotic inducers. All treatments significantly improved growth parameters in plants infected with *F. oxysporum* (Table 5). The most successful treatment was GF19-1 (SL, seedling treatment) in the plant height and the number of leaves in both seasons. The treatment with GF18-3 (SL, seedling treatment) was the best for increasing fresh and dry weights/plant in comparison to their control in both seasons. However, in the case of *R. solani* infection, the most useful treatment was GF18-3 as seedling treatment in increasing plant height, the number of leaves, fresh and dry weights/plant compared with untreated control in both seasons (Table 6).

#### 3.4.2. Effect of the Biological Inducers on the Activation of Defense Enzymes

Significant differences were found in phenol, peroxidase and polyphenol oxidase (Table 7). Application of GF19-1 as seedling treatment produced the highest enzyme values (peroxidase and polyphenol oxidase) and total phenols in case of *F. oxysporum*. However, in case of *R. solani*, the highest activities of peroxidase, polyphenol oxidase and total phenols were achieved by the treatments GF19-1 and GF18-3 as seedling treatment. Control treatment (pathogen only) showed the lowest values.

#### 3.4.3. Effect of Induction Treatments on the Relative Expression of PR4 Gene

**Figure 6:**
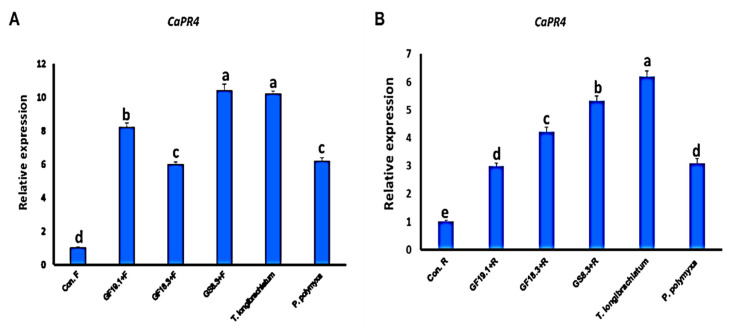
Real-time quantitative PCR analysis of the expression of *CaPR4* gene in control and treated pepper plants under infection with *Fusarium oxysporum* f.sp. *capsici* (**A**) and *Rhizctonia solani* (**B**). According to Fisher’s LSD, different letters denote significant differences.

Our results revealed a significant (*p ≤* 0.05) increase of *CaPR4* gene expression level in treated pepper plants compared to the control. Plants treated with GS8-3 showed the highest expression levels in the case of *F. oxysporum* and *R. solani*. In addition, plants infected with *F. oxysporum* and treated with GF18-3 resulted in the lowest up-regulation of *CaPR4*. However, under the infection with *R. solani*, treatment with GF19-1 presented the lowest gene expression (Figure 6).

## 4. Discussion

Pepper (*Capsicum annuum* L.) is a major commercial crop all over the world, with a huge societal and economic impact. It is one of Egypt’s most popular vegetable crops, cultivated either in open fields or in plastic greenhouses under a protected farming system. Pepper plants may be infected with various pathogens, including soil-borne pathogens [22]. In the greenhouse and fields of pepper plants, many fungal isolates from the genera *Fusarium*, *Macrophomina*, *Rhizoctonia*, *Verticilium*, *Pythium*, and *Sclerotinia* frequently cause damping-off, root rot, and wilt diseases [23,24]. Many researchers have observed that *Fusarium* wilt of pepper, caused by *Fusarium* spp., has resulted in significant reductions in pepper production in many countries around the world [22]. The use of a sustainable disease management strategy is necessary to reduce the impact of these diseases. Biological control is a long-term approach for disease management and healthy pepper production. *P. polymyxa* and *T. longibrachiatum* were isolated and identified by morphological and molecular biology techniques. Similarly, *P. putida* strain F1 was successfully identified based on the sequence of 16S rDNA comparing the sequence similarities with the related bacterial species in Gene bank [12]. In terms of antagonistic ability, the obtained findings showed that all of the tested bio-agents could significantly decrease the linear growth of *F. oxysporum* f.sp. *capsici* and *R. solani*. The results are in agreement with Sahii and Khalid [25], who found that the mycelial growth of *F. oxysporum* was hampered as a response to the antagonistic effect of *Trichoderma* sp. The *R. solani* mycelial diameter, as well as infection with root rot and damping-off diseases, were substantially decreased by *T. harzianum* isolate [26].

Many researchers have been interested in biological control mechanisms in recent decades. Pathogens are directly challenged by bioagents via hyperparasitism, antibiotic synthesis, and lytic enzyme production, as well as indirectly through competition for space and nutrients, developing systemic resistance, and encouraging plant development [27]. Using a scanning electron microscope, we discovered overgrowth and lysis of *F. oxysporum* f.sp. *capsici* and *R. solani* in dual cultures with *T. longibrachiatum*, as well as morphological anomalies such as atrophy and lysis using *P. polymyxa* in both fungal mycelia. Parasitism of pathogen fungi was reported by *Trichoderma* species in other studies [13]. Scanning electron microscopic analysis revealed that *T. harzianum* strains antagonist with *R. solani* [13]. *T. harzianum* Th-9 isolate overgrew and coiled around the *R. solani* cells, invading and damaging the host hypha. Through the mechanical activity, the host cells are penetrated. Secretion of antifungal compounds has been found to prevent the growth of different plant pathogens [7].

Plant growth promoting fungi (PGPF) is a kind of saprophyte that lives in the soil and promotes plant development. As a consequence of seed and seedling treatments, all of the evaluated biocontrol agents substantially decreased the severity of *Fusarium* wilt and root rot caused by *F. oxysporum* f.sp. *capsici* and *R. solani* compared to the control treatment. In several plants–PGPF combinations, colonization of roots with PGPF leads to a state of resistance in the whole plant known as induced systemic resistance. ISR in different plant species was introduced such as *Arabidopsis thaliana*, cucumber, and tobacco by PGPF application [6]. Curiously, PGPF isolates of *Penicillium simplicissmum* GP17-2, *Trichoderma asperellum* SKT-1, *Phoma* sp. GS 8-3, *F. equiseti* GF18-3, and *Phoma* sp. GS8-1 were highly effective in reducing the disease severity of white rot disease of onions [9]. In this study, the protective method of both types of biocontrol agents as individual treatments resulted in a substantial decrease in the disease. Cucua et al. [28] evaluated the efficacy of two biological control agents (BCAs) in suppressing *F. oxysporum* f.sp. *lycopersici* (*Bacillus subtilis* QST 713 and *Trichoderma* spp. TW2). Additionally, *P. polymyxa* NSY50 application on cucumber plants infected with *F. oxysporum* successfully decreased the incidence of *Fusarium* wilt [29]. In this study, the increase of phenolics and PR-Proteins such as peroxidase (PO), and polyphenoloxidase (PPO) inside pepper roots may have helped to limit *F. oxyporum* and *R. solani* infections. The accumulation levels of defense enzymes and the transcription levels of *PR1* and *PR5* genes were increased in cucumber plants treated with *T. atroviride* (TRS25) and led to better resistance against *R. solani* [30]. The fact that induction treatments substantially increased *CaPR4* gene expression suggests that this gene is involved in systemic resistance to *F. oxysporum* and *R. solani*. JA and ET activated *PR4*, *PR5*, and *PDF1*.2 in a synergistic manner [6]. Induced systemic resistance mediated by *P. simplicisimum* GP17-2 in *Arabidopsis* and tobacco enhanced the expression of different pathogenesis-related genes [6].

All treatments significantly enhanced growth characters in plants relative to the control infected with *F. oxysporum* and *R. solani*. PGPF has been shown to improve plant growth and disease control [6]. Several studies showed that *Trichoderma* isolates were considered proper biofertilizers, since they could improve the capacity of nutrients uptake in plants and the resistance toward plant pathogen [9]. Furthermore, *P. polymyxa* NMA1017 promoted plant growth through nitrogen fixation and siderophore synthesis, which led to increased crop production [31].

## 5. Conclusions

There is a growing request for safe biocontrol agents to improve management strategies of soil-borne diseases to reduce the poisonous effects of pesticides. The results indicated that the antagonistic isolates from healthy pepper plants and PGPF isolates can effectively control the pathogens, *F. oxysporum* f.sp. *capsici* and *R. solani*, as well as increasing growth parameters in pepper plants. The activities of oxidative enzymes (Peroxidase and polyphenol oxidase), phenol contents, and the expression levels of *CaPR4* were stimulated in the treated pepper plants, leading to induced resistance against *F. oxysporum* f.sp. *capsici* or *R. solani*. The ability to use these treatments to manage root rot and wilt diseases of pepper was already improved as a result of increased pepper growth.

## Figures and Tables

**Table 1:** Degree of antagonism of *Trichoderma* isolates against the linear growth of the pathogens *Fusarium oxysporum* f.sp. *capsici* and *Rhizoctonia solani*.

Antagonistic Isolates	Relative Power of Antibiosis (RPA) against *F. oxysporum*	RPA against *R. solani*
Control	0.000 g *	0.000 h
*Paenibacillus polymyxa* (B25)	1.663 a	1.260 a
*Bacillus subtilis* (B17)	0.933 c	0.780 c
*Bacillus subtilis* (B9)	0.960 b	0.098 g
*Bacillus thuringiensis* (B11)	0.910 d	0.860 b
*Bacillus subtilis* (B12)	0.886 e	0.150 f
*Bacillus subtilis* (B31)	0.216 f	0.390 e

* According to Fisher’s LSD, different letters denote significant differences.

**Table 2:** Effect of different bacterial antagonists on radial growth of the pathogens *Fusarium oxysporum* f.sp. *capsici* and *Rhizoctonia solani*.

*Trichoderma* Isolate Code No.	Values of Antibiosis against *F. oxysporum*	Values of Antibiosis against *R. solani*
*Trichoderma longibrachiatum* (T1)	1	1
*Trichoderma aureoviride* (T2)	2	2
*Trichoderma hamatum* (T3)	2	2
*Trichoderma harzianum* 1 (T4)	3	2
*Trichoderma harzianum* 2 (T5)	3	3

**Table 3:** Effect of the tested bioagents on the severity (%) of wilt/root rot of pepper plants caused by *Fusarium oxysporum* f.sp. *capsici* and *R. solani* under greenhouse conditions.

Treatment	*F. oxysporum* f.sp. *capsici*		*R. solani*	
	Season1 (S1)	Season2 (S2)	Season1	Season2
Control	90.27 a *	83.32 a	92.58 a	87.02 a
GF 19-1 (SS)	31.94 bcd	29.16 bcde	35.18 bc	35.18 bc
GF 18-3 (SS)	31.94 bcd	29.16 bcde	33.33 c	33.33 bc
GS 8-3 (SS)	34.71 b	33.33 b	35.18 bc	35.18 bc
GF 19-1 (SL)	27.77 e	25.00 e	33.33 c	31.47 cd
GF 18-3 (SL)	29.16 de	26.38 de	27.77 d	27.77 d
GS 8-3 (SL)	33.33 bc	30.55 bcd	33.33 c	33.33 bc
*P. polymyxa* (SS)	33.33 bc	30.55 bcd	38.88 b	37.03 b
*T. longibrachiatum* (SS)	31.94 bcd	31.94 bc	33.33 c	35.18 bc
*P. polymyxa* (SL)	29.16 de	27.77 cde	33.33 c	33.33 bc
*T. longibrachiatum* (SL)	30.55 cde	29.16 bcde	31.47 cd	35.17 bc

(SS) = seed soaking, (SL) = seedling treatment, GF19-1 = *Fusarium equiseti* GF19-1, GF18-3 = *Fusarium equiseti* GF18-3, GS8-3 = *Phoma* sp. GS8-3, *P. polymyxa* = Paenibacillus polymyxa, *T. longibrachiatum* = Trichoderma longibrachiatum. * According to Fisher’s LSD, different letters denote significant differences.

**Table 4:** Effect of different bioagent on discoloration severity (%) of vascular bundles in roots of pepper plants infected with the fungus *Fusarium oxysporum* f.sp. *capsici* as an indicator of the disease severity.

Treatment	Discoloration Severity %	
	Season1	Season2
Control	74.06 a *	77.77 a
GF19-1 (SS)	22.22 bc	18.51 b
GF-3 (SS)	22.21 bc	18.51 b
GS 8-3 (SS)	22.22 bc	22.22 b
GF19-1 (SL)	14.81 c	14.81 b
GF18-3 (SL)	14.81 c	18.51 b
GS 8-3 (SL)	22.22 bc	18.51 b
*P. polymyxa* (SS)	22.22 bc	22.22 b
*T. longibrachiatum* (SS)	25.92 b	22.22 b
*P. polymyxa* (SL)	18.51 bc	18.51 b
*T. longibrachiatum* (SL)	22.22 bc	18.51 b

(SS) = seed soaking, (SL) = seedling treatment, GF19-1 = *Fusarium equiseti* GF19-1, GF18-3 = *Fusarium equiseti* GF18-3, GS8-3 = *Phoma* sp. GS8-3, *P. polymyxa* = *Paenibacillus polymyxa*, *T. longibrachiatum* = *Trichoderma longibrachiatum.* * According to Fisher’s LSD, different letters denote significant differences.

**Table 5:** Effect of different bioagent on some plant growth parameters of pepper plants infected with *Fusarium oxysporum* f.sp. *capsici* under greenhouse conditions.

Treatment	Plant Height (cm)		No. Leaves/Plant		Shoot Weight (g)			
					Fresh		Dry	
	S1	S2	S1	S2	S1	S2	S1	S2
Control	17.33 f *	15.83 e	8.66 d	8.00 d	6.10 g	6.16 g	2.03 d	2.24 f
GF 19-1 (SS)	31.33 ab	30.33 b	15.66 b	15.33 abc	9.13 c	9.16 cd	3.83 b	4.13 b
GF18-3 (SS)	30.53 bcd	29.86 bc	16.66 a	15.66 ab	10.16 b	10.36 b	4.70 a	4.83 a
GS 8-3 (SS)	28.33 cde	26.70 d	15.33 b	14.66 bc	8.90 d	8.95 d	3.86 b	4.10 b
GF 19-1 (SL)	33.33 a	32.50 a	16.66 a	16.33 a	10.33 b	10.44 b	4.83 a	4.66 a
GF 18-3 (SL)	31.00 abc	31.16 ab	15.66 b	15.33 abc	11.10 a	11.16 a	4.93 a	4.86 a
GS 8-3 (SL)	29.66 bcde	28.50 c	15.66 b	15.33 abc	9.16 c	9.22 c	3.66 b	3.74 c
*P. polymyxa* (SS)	28.16 de	26.16 d	14.33 c	14.33 c	7.03 ef	7.23 ef	2.93 c	3.13 e
*T. longibrachiatum* (SS)	27.66 e	26.00 d	15.66 b	15.33 abc	6.90 f	7.14 f	2.90 c	3.05 e
*P. polymyxa* (SL)	28.33 cde	25.83 d	15.33 b	14.66 bc	7.03 ef	7.27 ef	3.03 c	3.36 d
*T. longibrachiatum* (SL)	30.83 abcd	26.60 d	16.00 ab	15.66 ab	7.23 e	7.46 e	3.13 c	3.41 d

(SS) = seed soaking, (SL) = seedling treatment, GF19-1 = *Fusarium equiseti* GF19-1, GF18-3 = *Fusarium equiseti* GF18-3, GS8-3 = *Phoma* sp. GS8-3, *P. polymyxa* = *Paenibacillus polymyxa*, *T. longibrachiatum* = *Trichoderma longibrachiatum.* * According to Fisher’s LSD, different letters denote significant differences.

**Table 6:** Effect of different bioagents on some plant growth parameters of pepper plants infected with *R. solani* under greenhouse conditions.

Treatment	Plant Height (cm)		No. Leaves/Plant		Shoot Weight (g)			
					Fresh		Dry	
	S1	S2	S1	S2	S1	S2	S1	S2
Control	16.83 g *	17.33 g	8.66 e	9.00 c	5.83 g	6.16 g	1.66 f	1.80 f
GF 19-1 (SS)	28.50 c	29.00 c	14.00 bcd	15.00 b	9.66 c	9.83 cd	3.13 cd	3.43 c
GF 18-3 (SS)	28.83 c	29.33 c	15.66 ab	16.00 ab	10.66 b	10.93 b	4.16 ab	4.36 b
GS. 8-3 (SS)	26.50 de	27.83 d	13.66 cd	14.66 b	8.83 d	9.16 d	3.20 c	3.50 c
GF 19-1 (SL)	29.83 b	30.33 b	14.66 bcd	16.00 ab	10.73 b	10.90 b	4.00 b	4.33 b
GF 18-3 (SL)	32.00 a	33.00 a	16.66 a	17.33 a	11.73 a	11.80 a	4.33 a	4.76 a
GS. 8-3 (SL)	26.66 d	27.66 d	15.33 abc	15.66 ab	9.80 d	9.96 c	3.13 cd	3.26 cd
*P. polymyxa* (SS)	25.66 e	26.66 e	13.33 d	14.33 b	6.66 f	7.16 f	2.80 e	2.90 e
*T. longibrachiatum* (SS)	24.70 f	25.33 f	14.66 bcd	15.33 b	6.26f g	6.60 fg	2.76 e	2.86 e
*P. polymyxa* (SL)	25.66 e	27.16 de	14.66 bcd	15.66 ab	6.76 f	6.86 fg	2.83 e	2.90 e
*T. longibrachiatum* (SL)	26.66 d	27.33 de	15.66 ab	16.00 ab	7.80 e	8.06 e	2.90 de	3.06 de

(SS) = seed soaking, (SL) =seedling treatment, GF19-1 = *Fusarium equiseti* GF19-1, GF18-3 = *Fusarium equiseti* GF18-3, GS8-3 = *Phoma* sp. GS8-3, *P. polymyxa* = *Paenibacillus polymyxa*, *T. longibrachiatum* = *Trichoderma longibrachiatum.* * According to Fisher’s LSD, different letters denote significant differences.

**Table 7:** Effect of different bioagents on enzyme activities and phenol contents in plants infected with *Fusarium oxysporum* f.sp. *capsici* and *Rhizoctonia solani*.

Treatment	*Fusarium*			*Rhizoctonia*		
	Phenol	POX	PPO	Phenol	POX	PPO
Control	0.327 i *	0.420 j	0.156 j	0.376 f	0.483 g	0.156 i
GF 19-1 (SS)	0.624 e	0.840 b	0.420 d	0.636 c	0.776 bc	0.366 e
GF 18-3 (SS)	0.683 cd	0.790 d	0.402 e	0.650 c	0.786 b	0.370 e
GS 8-3 (SS)	0.516 h	0.763 e	0.320 h	0.566 d	0.767 cd	0.346 f
GF 19-1 (SL)	0.756 a	0.860 a	0.484 a	0.696 ab	0.826 a	0.440 b
GF 18-3(SL)	0.713 b	0.826 c	0.423 c	0.713 a	0.836 a	0.460 a
GS 8-3 (SL)	0.673 d	0.743 f	0.397 f	0.516 e	0.746 d	0.383 d
*P. polymyxa* (SS)	0.541 g	0.544 i	0.283 i	0.576 d	0.626 f	0.313 h
*T. longibrachiatum* (SS)	0.673 d	0.700 h	0.373 g	0.656 c	0.753 d	0.406 c
*P. polymyxa* (SL)	0.584 f	0.721 g	0.420 d	0.563 d	0.656 e	0.336 g
*T. longibrachiatum* (SL)	0.690 c	0.822 c	0.441 b	0.686 b	0.746 d	0.403 c

(SS) = seed soaking, (SL) = seedling treatment, GF19-1 = *Fusarium equiseti* GF19-1, GF18-3 = *Fusarium equiseti* GF18-3, GS8-3 = *Phoma* sp. GS8-3, *P. polymyxa* = *Paenibacillus polymyxa*, *T. longibrachiatum* = *Trichoderma longibrachiatum.* * According to Fisher’s LSD, different letters denote significant differences.

## Data Availability

Not applicable.
